# Data analysis of patients with positive mould or dimorphic fungal cultures from sterile sites

**DOI:** 10.4102/sajid.v39i1.630

**Published:** 2024-08-30

**Authors:** Bonita van der Westhuizen, Samantha Potgieter

**Affiliations:** 1 Department of Medical Microbiology, Faculty of Health Sciences, University of the Free State, Bloemfontein, South Africa; 2 Department of Medical Microbiology, National Health Laboratory Service, Bloemfontein, South Africa; 3 Department of Internal Medicine, Faculty of Health Sciences, University of the Free State, Bloemfontein, South Africa

**Keywords:** fungal infection, distribution, isolates, risk factors, treatment, outcome

## Abstract

**Background:**

Moulds and dimorphic fungi are increasingly recognised as pathogens carrying high morbidity and mortality in critically ill and immune-compromised patients. The lack of surveillance data limits our understanding of these infections.

**Objectives:**

To determine the distribution, patient characteristics, risk factors, therapy and treatment outcome in patients with positive mould or dimorphic fungal cultures from sterile sites at a tertiary hospital in central South Africa.

**Method:**

All moulds or dimorphic fungi cultured from sterile specimens from 1 July 2014 to 30 June 2017 were identified retrospectively. Laboratory and clinical records were reviewed. Information collected included gender and age, type of specimen collected for investigation, specific fungi isolated, underlying conditions, other contributing risk factors, treatment and outcome of the patients.

**Results:**

Forty-eight patient records were analysed. Male and female patients were equally distributed. The mean age was 40.5 years (range 7–78 years). *Aspergillus* spp. were most commonly isolated. The most common underlying condition was HIV infection, followed by haematological conditions. Twenty-six (54.2%) patients received treatment involving antifungal therapy alone (*n* = 19; 73.1%), surgery alone (*n* = 5; 19.2%) or a combined medical and surgical approach (*n* = 2; 7.7%). Twenty-two (45.8%) patients received no treatment. The overall mortality rate was 25.0% (*n* = 12).

**Conclusion:**

The diagnosis of fungal infections remains challenging. In the current study, moulds and dimorphic fungi were isolated from at-risk patients’ specimens. Despite treatment with appropriate antifungal agents, the associated mortality rate was high.

**Contribution:**

This study contributes to the growing body of knowledge on these potentially life-threatening infections.

## Introduction

Fungi, including moulds, are increasingly recognised as important pathogens in critically ill and immune-compromised patients.^1,2^ Not only have these organisms assumed a greater role in human disease over the past two decades, but invasive fungal infections, and in particular mould infections, are associated with significant morbidity and mortality.^3,4^

Despite general agreement that mould infections are becoming more important, our understanding of these diseases remains incomplete, mainly because of the lack of surveillance data. The most commonly isolated moulds in international studies are *Aspergillus* spp., *Fusarium* spp. and mucoraceous moulds.^1,5,6,7^ In a study conducted in KwaZulu-Natal, South Africa, *Aspergillus* spp. were the most commonly isolated moulds among critically ill children.^8^ Previous South African studies have reported an increase in HIV-positive patients presenting with an unmasking immune reconstitution inflammatory syndrome (IRIS), with infections caused by *Emergomyces africanum* (formerly known as *Emmonsia* spp.).^9,10^ Fungal infections associated with HIV infection are endemic to the Western Cape Province in South Africa, with the most common species presenting with skin lesions identified as *E. africanum, Histoplasma capsulatum* and *Sporothrix schenckii*.^11^ More recent evidence highlighted severe adult respiratory syndrome (SARS) associated with coronavirus (CoV-2) infection (coronavirus disease 2019 [COVID-19]) as a new risk factor for fungal infections, especially infections caused by *Aspergillus* spp. and the mucoraceous moulds.^12^ Except for these publications, limited local data regarding mould infections are available, most likely because of diagnostic challenges.

With a retrospective review of laboratory and patient data, this study aimed to determine the distribution, patient characteristics, risk factors, therapy and treatment outcome in patients with positive mould or dimorphic fungi cultured from sterile sites at Universitas Academic Hospital (UAH) in the Free State Province, South Africa.

## Research methods and design

### Study design and setting

A retrospective, observational descriptive study was conducted. Patients admitted to UAH between 01 July 2014 and 30 June 2017, in whom a mould or dimorphic fungus was isolated from specimens sent to the laboratory for fungal culture, were included in the study. Universitas Academic Hospital is the only tertiary referral hospital providing specialist and sub-specialist level care for the Free State and Northern Cape provinces.

All culture-positive moulds and dimorphic fungi from sterile sites (tissue specimens, blood cultures, peritoneal fluid and cerebrospinal fluid) were included in the final analysis. Although bronchoalveolar lavage fluid, endotracheal aspirates and sputum specimens are not considered sterile specimens, they were still included in the study if the same organism was cultured from a second specimen, and the patient had symptoms of an invasive fungal disease of the lungs together with supporting clinical and/or radiological signs. A patient was considered to have symptoms in keeping with a fungal infection when the presence of a cough, fever, dyspnoea, haemoptysis or chest pain was documented in the clinical notes. A patient was considered to have radiological features in keeping with a fungal infection when an imaging study reported by a consultant radiologist was consistent with the diagnosis of a possible fungal infection.

We excluded all specimens that yielded a yeast, specimens with a mould isolated from a non-sterile site (except for selected respiratory specimens) and non-human specimens.

### Sample analysis

Isolates were cultured at the Universitas National Health Laboratory Service (NHLS) Microbiology Laboratory, a SANAS (South African National Accreditation System) accredited laboratory. All specimens had been processed according to the standard operating procedure of the laboratory.^13^ Specimens were inoculated onto two Sabouraud-Dextrose agar plates and incubated at 25°C and 37°C, respectively, for 14 days. The plates were examined daily for fungal growth. When growth was observed, the macroscopic appearance of the isolates was described, and a lactophenol cotton blue stain was performed for the microscopic examination to determine the identity of the fungus. The fungi that proved difficult to identify in our laboratory were sent to the mycology reference laboratory at the National Institute of Communicable Diseases (NICD) for an in-house broad-range fungal polymerase chain reaction (PCR). Histology results were reviewed for all samples submitted for histological evaluation.

### Data collection

Laboratory results were obtained from the Central Data Warehouse of the NHLS. All specimens registered for mycology culture at UAH for the period 01 July 2014 to 30 June 2017 were reviewed. Patient information was collected from the electronic patient file system, Meditech, used at UAH.

Patients’ clinic and hospital files were used when the electronic information was not available. Information collected included gender and age, type of specimen collected for investigation, specific mould or dimorphic fungus isolated, symptoms, radiological investigations, underlying conditions, other contributing risk factors, treatment and outcome of the patients. Outcome was defined as either improved, unchanged, in-hospital death or unknown at 12 weeks after sample collection. Patients for whom little or no clinical data were available were excluded from the study. With the assistance of an infectious diseases specialist physician, data were captured on a Microsoft Excel (version 2016) spreadsheet approved by the Department of Biostatistics of the University of Free State (UFS).

### Data analysis

Continuous variables were summarised by medians, minimum, maximum or percentiles. Categorical variables were summarised by frequencies and percentages. Differences between groups were evaluated using appropriate statistical tests (chi-square or Fisher’s exact test) for unpaired data. The analysis was performed by the Department of Biostatistics, UFS, using SAS version 9.4 (SAS Institute Inc.; Cary, NC, USA).

### Ethical considerations

Permission to conduct the study was obtained from the NHLS business manager, the acting Head of the Department of Medical Microbiology and the Free State Province Department of Health. Ethical approval was granted by the Health Sciences Research Ethics Committee (HSREC) of the University of the Free State (ethics number: UFS-HSD2017/1122). Because data were collected from archived patient and laboratory records, informed consent was not required. Confidentiality was ensured by allocating a number to each patient’s record and excluding all personal information.

## Results

**FIGURE 1:**
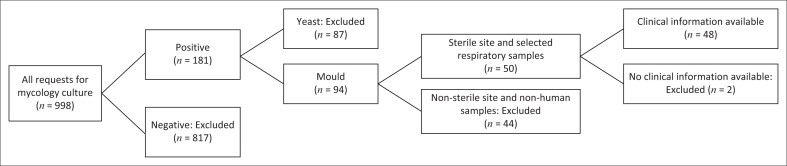
Selection of records of patients with invasive mould infections.

From a total of 998 samples submitted for mycology culture, 950 were excluded based on the exclusion criteria, as illustrated in Figure 1. In total, 48 isolates were included in the final analysis.

### Clinical features

The patients’ gender and underlying conditions are presented in Table 1. Male and female patients had equal numbers of positive samples. The median age was 40.5 years (range 7–78 years). The most common underlying clinical condition was HIV (*n* = 14; 29.2%), while only 10.4% (*n* = 5) of patients had no identifiable risk factors. The median CD4 count in the HIV- positive patients was 88.5 cells/µL (range 1 cells/µL – 568 cells/µL). There were no patients that received bone marrow or solid organ transplants and no patients fulfilled the criteria for prolonged intensive care unit (ICU) stay defined as > 7 days.

**TABLE 1:** Patients’ gender, underlying clinical conditions and outcome (*n* = 48).

Variables	*n*	%
**Gender**		
Male	24	50.0
Female	24	50.0
**Underlying condition**		
HIV	14	29.2
Haematological malignancy	9	18.8
Neutropenia	8	16.7
Chemotherapy	8	16.7
Chronic kidney disease on peritoneal dialysis	8	16.7
Solid organ malignancy	3	6.3
Structural lung disease	3	6.3
Diabetes mellitus	2	4.2
Aplastic anaemia	2	4.2
Allergic rhinosinusitis with nasal polyps	2	4.2
Steroid therapy	1	2.1
Primary immunodeficiency	1	2.1
Myeloproliferative disease	1	2.1
Ventriculo-peritoneal shunt	1	2.1
None identified	5	10.4
**Outcome**		
Improved	32	66.7
Unchanged	1	2.1
Demised in hospital	12	25.0
Unknown†	3	6.3

† , Lost due to follow-up or information not recorded.

### Laboratory findings

Table 2 summarises specimen distribution of the culture-positive samples as well as the mould species isolated. Tissue was the most common sample type from which a mould was isolated (*n* = 29; 60.4%), followed by respiratory samples and peritoneal dialysis fluid. Tissue samples consisted of skin and mucus membrane specimens. The presence of a fungal infection was confirmed upon histological examination in 37.9% (*n* = 11) of tissue samples. In 20.7% (*n* = 6) of the tissue specimens, histological evaluation was not requested. *Aspergillus* spp. was by far the most commonly isolated mould (*n* = 19; 39.6%), followed by *Fusarium* spp., *Bipolaris* spp. and the mucoraceous moulds.

**TABLE 2:** Type of specimens received by the laboratory for mycology culture and mould species isolated.

Type of specimens	*n*	%
**Type of specimens (*n* = 48)**		
Tissue	29	60.4
Skin	18	62.1
Mucus membrane	11	37.9
Respiratory specimens	8	16.7
Sputum	3	37.5
Bronchial washing	2	25.0
Endotracheal aspirate	3	37.5
Peritoneal fluid	8	16.7
Cerebrospinal fluid	2	4.2
Blood culture†	1	2.1
**Species isolated (*n* = 48)**		
*Aspergillus* spp.	19	39.6
*Fusarium* spp.	5	10.4
*Bipolaris* spp.	5	10.4
Mucoraceous moulds	4	8.3
*Cladosporium* spp.	3	6.3
*Sporothrix schenckii*	3	6.2
*Penicillium* spp.	2	4.2
*Alternaria* spp.	2	4.2
*Histoplasma capsulatum*	1	2.1
*Neurospora* spp.	1	2.1
*Chaetomium* spp.	1	2.1
*Phoma spp.*	1	2.1
*Emmonsia* spp.	1	2.1

† , *Neurospora* spp. was isolated from the positive blood culture.

It should be noted that although the tissue samples that were negative for fungal growth were not included in this study for further analysis, roughly 70% of these were also submitted for histological examination. Upon histological examination, 40% of these tissue samples were negative for fungal elements, approximately 10% were positive for invasive fungal elements and the remainder were suggestive of other pathology.

The fungal species isolated from the 14 HIV-positive patients were *Sporothrix schenckii, Bipolaris* spp., *Aspergillus* spp., *H. capsulatum, E. africanum, Penicillium* spp., *Cladosporium* spp. and *Saksenaea oblongispora*. A tissue sample from one patient with rhino-orbital-cerebral mucormycosis yielded *S. oblongispora* as confirmed by PCR. The most common mould species isolated from the 12 patients with haematological conditions were *Aspergillus* spp. 33.3% (*n* = 4). The majority of patients with haematological conditions were neutropenic (*n* = 8; 66.7%) at the time of culture collection, and two (16.2%) patients were known to be HIV-positive.

### Treatment

The treatment modalities chosen for each patient are summarised in Table 3. Of the 26 patients receiving any form of treatment, 19 (73.1%) were treated with antifungal therapy alone. The majority (*n* = 15; 78.9%) of these patients received combination therapy, mostly amphotericin B deoxycholate (ABD) combined with an azole. In seven (26.9%) patients, surgical intervention was performed, with two of these patients also receiving additional antifungal therapy. Five patients were treated surgically only, of which four were diagnosed with nasal polyps and chronic allergic fungal rhinosinusitis. The fifth patient was diagnosed with a pulmonary aspergilloma. Twenty-two (45.8%) patients received no treatment.

**TABLE 3:** Summary of treatment results.

Treatment options	*n*	%
**Treatment received (*n* = 48)**		
Yes	26	54.2
No†	22	45.8
**Treatment modality (*n* = 26)**		
Antifungal therapy alone	19	73.1
Surgery alone	5	19.2
Surgery plus antifungal therapy	2	7.7
**Antifungal agents (*n* = 21)** ‡		
Amphotericin B deoxycholate (ABD)	17	81.0
Fluconazole	7	33.3
Itraconazole	7	33.3
Voriconazole	5	23.8
Terbinafine	1	4.8

† , Death before treatment was initiated (*n* = 3), pulmonary aspergilloma managed conservatively (*n* = 1), and the mould isolated regarded as contamination (*n* = 18).

‡ , The total of the n-values exceeds 21 as some patients were treated with a combination of antifungal agents.

The number, specimen type, fungi isolated, and whether supporting histology results were available in the 26 patients that received any form of therapy are presented in Table 4.

**TABLE 4:** Specimen types, organisms isolated and supporting histology performed.

Specimens		Organisms isolated		Supporting histology (*n*)	Outcome (*n*)		
Specimen type	*n*	Organism name	*n*		Demised	Improved	Unknown
**Respiratory specimens**	6			N/A	2	4	-
Endotracheal aspirate	3	*Aspergillus* spp. *Penicillium* spp.	5 1				
Bronchoalveolar lavage	1						
Sputum	2						
**Mucus membrane tissue**	10			6	2	8	-
Nasal	7	*Rhizopus* spp. *Aspergillus* spp. *Bipolaris* spp. *Alternaria* spp. *Phoma* spp.	2 3 3 1 1				
Palate	1						
Lip	1						
Maxillary sinus	1						
***Skin tissue***	8	*Sporothrix shenkii* *Aspergillus* spp. *Emergomyces africanum* *Histoplasma capsulatum* *Bipolaris* spp.	3 2 1 1 1	5	1	5	2
**Blood culture**	1	*Neurospora* spp.	1	N/A	1	-	-
**Joint tissue**	1	*Cladosporium* spp.	1	†	-	1	-

N/A, Sample type not suitable for histological evaluation..

† , Not sent for histological evaluation.

In 18 of the 22 patients that received no treatment, the isolated mould was considered a contaminant. In eight of these patients, a mould was isolated from peritoneal dialysis (PD) fluid and considered a contaminant based on additional biochemical and microbiological results. In another eight of these patients, an alternative diagnosis was confirmed on histology and the mould was considered a contaminant. In the additional two patients where the mould was not deemed significant, the decision was based on clinical grounds. Of the remaining four patients that received no therapy, three demised before treatment could be initiated and one patient was diagnosed with pulmonary aspergillomas not suitable for surgery.

### Outcome

Table 4 outlines the patient outcomes in this study. Most of the patients (*n* = 32; 66.7) had a favourable outcome. However, the in-hospital mortality rate was 25% (*n* = 12). No statistically significant difference regarding the mortality rate among the treated (*n* = 6/26; 23.1%) and untreated (*n* = 6/22; 27.3%) patients was observed (*p* = 0.31).

In three of the six patients that demised in the untreated group, the mould was considered a contaminant as histological evaluation reported malignant cells and no fungal invasion. The remainder of the patients not receiving therapy showed improvement, except for one patient with multiple, bilateral aspergillomas that remained in an unchanged condition.

In the group of patients treated with antifungal agents and/or surgery, 23.1% demised during hospital stay. All of these patients had serious risk factors including aplastic anaemia, haematological malignancy and primary immunodeficiency. Mucoraceous moulds and *Aspergillus* spp. were the most common isolates in this group. These patients demised despite appropriate antifungal therapy with ABD. All five patients that were treated with surgery alone had favourable outcomes.

## Discussion

The diagnosis of fungal infections remains a challenge, as the clinical manifestations are often non-specific. A lack of reliable diagnostic testing makes it difficult to estimate the true burden of fungal disease.^6^ The reported sensitivity of histopathological methods for diagnosing invasive fungal infections is approximately 78%, as compared to 8% – 60% for culture.^14,15^

In keeping with the findings of international studies, we also found that *Aspergillus* spp., followed by *Fusarium* spp., *Bipolaris* spp. and the mucoraceous moulds, were the most common fungi isolated.^1,5,6,7^ Enoch et al.^1^ reported that the most common mould isolated in the UK is *Aspergillus* spp. However, *Fusarium* spp., *Scedosporium* spp., *Penicillium* spp. and the Mucorales occur at an increasing rate.^1^ Malani et al. reported a similar scenario in the USA.^7^

Risk factors for developing fungal infections include prolonged ICU stay, solid organ transplants, haematopoietic stem cell transplants, haematological malignancies, neutropenia, burn wounds, HIV infection, invasive medical devices, and grafts. The use of antineoplastic and immunosuppressive agents, broad-spectrum antibiotics, and more aggressive surgery has also been identified as important contributing factors.^1,4,5,16^ The risk factors identified in our study population generally reflect those reported by others.

In our study, we identified HIV with a median CD4 count of 88.5 cells/µL as an important risk factor, being present in 29.2% (*n* = 14) of cases, a finding that has not previously been well-documented. This may be because of the paucity of data from countries with a high burden of HIV infection. It should be noted that the background prevalence of HIV in the Free State Province during our study period was estimated at 5.1% as released in the mid-year population estimates for 2018 by Statistics South Africa.^17^ *Saksenaea oblongispora* has recently been recognised as an emerging Mucorales.^18^ To our knowledge, this is the first case of invasive *S. oblongispora* infection documented in the setting of HIV.

The most common mould species isolated from the patients with haematological conditions were *Aspergillus* spp., which was in keeping with findings reported in the literature. Studies have also shown that there is an increase in infections with the Mucorales, *Fusarium* spp. and *Bipolaris* spp.^19^ We made similar findings with the mucoraceous moulds being the second most common fungal isolate in this patient group.

The different combinations of antifungal drugs varied widely between the patients, and therefore, it is not possible to draw any further conclusions from the different combinations selected. These findings reaffirm the challenges that clinicians face, not only regarding confirmation of the diagnosis of fungal infections, but also in choosing the most appropriate antifungal therapy. Consultation with an infectious diseases specialist or microbiologist should therefore be considered for all patients with suspected invasive mould infections. Studies have reported improvement in antifungal therapy use as well as appropriate testing and follow-up in patients for whom an infectious diseases specialist consultation was requested. These studies mainly included patients with candidemia,^20,21^ although one would expect the same results in the context of invasive mould infections.

The four patients diagnosed with nasal polyps all underwent surgical removal of the polyp. The currently available data and consensus guidelines do not support the use of antifungal agents in patients with chronic allergic rhinosinusitis.^22^ The patient diagnosed with a pulmonary aspergilloma underwent a fenestration procedure as suggested by the Infectious Diseases Society of America’s guidelines for the treatment of aspergillomas.^23^ All five patients had favourable outcomes, reflecting what is known about the management and outcome of these patients.

Only two patients were treated with a combination of antifungal therapy and a surgical intervention. The first patient had a haematological malignancy and developed a *Bipolaris* spp. fungal sinusitis. The patient received ABD and underwent repeated surgical debridement and had a favourable outcome. Another patient had *Cladosporium* spp. cultured from five different tissue samples collected intra-operatively during a wash-out procedure for a prosthetic joint infection. The patient was treated with fluconazole, multiple courses of broad-spectrum antibiotics, and improved clinically. It was noteworthy that this patient improved following aggressive debridement despite the lack of appropriate antifungal therapy. These cases highlight the important role for aggressive surgical intervention when required to ensure adequate source control.

In 18 patients, the isolated fungus was considered to represent contamination. This confirms the view that the mere isolation of a fungal agent does not equate to disease and should always be correlated with clinical findings to determine the significance of the isolates.^24,25^

The overall in-hospital mortality rate was 25.0%. A large multicenter study conducted in Asia found an overall mortality rate of 32.9%,^26^ which was notably higher than in our study. Data on the overall mortality rate of invasive fungal infections, inclusive of patients with all risk factors, are limited. The bulk of available data reports on the mortality in specific groups of patients with specific risk factors. The mortality rate among our patients was slightly higher in the untreated group compared to the treated group, although this difference was not statistically significant.

We observed a significantly higher mortality rate in patients with underlying haematological conditions compared to the HIV-positive group (*n* = 5/12 [41.7%] vs. *n* = 2/14 [14.3%]), reflecting data that have been reported in the literature. In the HIV-positive group, the two patients who demised had very low CD4 counts of 30 cells/µL and 50 cells/µL, respectively, nine had a favourable outcome, and three were lost to follow up. These three patients had CD4 counts of 1 cells/µL, 13 cells/µL and 198 cells/µL, respectively, were all started on antifungal therapy and referred back to their primary facilities. None attended their follow-up appointments. It is possible that these patients might have demised. Should this assumption be true, it would increase the mortality rate in the HIV-positive group to 35.7%. It should be noted that not only did these patients have different underlying risk factors, but the type of fungi identified was also very different, which may contribute to mortality.

A limitation of this study was the small sample size. Only sterile specimen types were included in our analysis. Therefore, we might have underestimated the true burden of mould and dimorphic fungal infections. Furthermore, the study was done retrospectively and most of the moulds were reported to genus level only. Specific durations of therapy and reasons for chosen regimens could not be elucidated from the patient files as record-keeping was poor in the majority of cases.

Fungal infections cause a high burden of disease in South Africa, driven largely by HIV, tuberculosis (TB) and poverty.^27^ We believe this study contributes to the growing body of knowledge on the distribution, patient characteristics and outcomes of fungal infections, particularly among patients in the Free State Province, and lays the foundation for further research in the field of medical mycology.

## Conclusion

The diagnosis of fungal infections remains a challenge. In the current study, moulds and dimorphic fungi were found to cause serious infections, especially in at-risk patients, with HIV found to be the most common risk factor in our setting. However, moulds are also common environmental organisms and frequently a source of contamination of clinical specimens, thus highlighting the need for clinical correlation in the interpretation of these results. Despite treatment with appropriate antifungal agents, the associated mortality rate of 25.0% was still high.
